# Erectile dysfunction in patients with anxiety disorders: a systematic review

**DOI:** 10.1038/s41443-020-00405-4

**Published:** 2021-02-18

**Authors:** Rajalaxmi Velurajah, Oliver Brunckhorst, Muhammad Waqar, Isabel McMullen, Kamran Ahmed

**Affiliations:** 1 GKT School of Medicine, Department of Bioscience Education, King’s College London, London, UK; 2 MRC Centre for Transplantation, Guy’s Hospital Campus, King’s College London, King’s Health Partners, London, UK; 3 Department of Urology, King’s College Hospital, London, UK; 4 South London and Maudsley NHS Foundation Trust, London, UK

**Keywords:** Erectile dysfunction, Sexual dysfunction, Psychology

## Abstract

Men with anxiety disorders have been identified as high risk of developing erectile dysfunction (ED). The aim of this review is to define the prevalence and severity of ED in the male anxiety disorder population. A literature search of three electronic databases (PubMed, Embase and PsychINFO) and a grey literature registry was conducted. Inclusion criteria were studies that investigated adult males, documented diagnosis of anxiety disorders made by a qualified psychiatrist and use of a validated tool to diagnose ED such as International Index of Erectile Function or ICD-10/DSM-IV. The search yielded 1220 articles and 12 studies were selected. The anxiety disorders investigated were post-traumatic stress disorder, obsessive–compulsive disorder, social phobia/social anxiety disorder and panic disorder. We found that the median [IQR] prevalence of ED was 20.0 [5.1–41.2]% and the median [IQR] International Index of Erectile Function-5 scores were 17.62 [13.88–20.88], indicating a mild to moderate severity. Our review suggests a high prevalence of ED in the anxiety disorder population and ED may be more severe in this cohort, therefore advocating this is an important clinical topic. However, the evidence is limited due to the high heterogeneity between the studies and more research is required in this field.

## Introduction

Erectile dysfunction (ED) is the inability to achieve or maintain a penile erection satisfactory for sexual intercourse [1]. ED has both organic and psychogenic causes; the mechanism of how psychogenic factors such as anxiety and depression leads to ED is not fully understood. Psychiatric illness has been associated with sexual dysfunctions in both men and women, this could be through the mental health disorder itself or the psychotropic medications used to treat them [2].

ED has a substantial effect on the quality of life and patients’ well-being. Many studies have shown that the psychological impact of ED is greater in men with greater erectile impairment [3]. Men with ED tend to have lower self-esteem and poorer satisfaction for sexual activity thereby making them prone to have anxiety and depression [3]. The presence of anxiety disorders in the ED population has been reported to be up to 37%, in addition, ED has also been associated with free-floating anxiety [4]. The role of anxiety in ED has not been clearly established, however, it is proposed that anxiety contributes to a vicious cycle that impairs the sexual relation between the patient and partner resulting in communication problems, which further impede sexual functioning [5]. It is also suggested that a small level of anxiety contributes to the physiologically normal sexual cycle especially during the arousal stages, this is thought to be because there is an overlap of features between arousal and the typical anxiety response such as tachycardia and excess sweating [6, 7].

There have been other reviews that look at ED and other mental conditions like depression [8], however, there has not been any review evaluating the association between anxiety and ED. Both ED and anxiety disorders have been underdiagnosed in primary care [9, 10]. This makes the patients suffering from these conditions at a higher risk of having a low quality of life, as they are less likely to be identified and be given the care and support they need. Therefore, this review was proposed to explore the link between these conditions.

This systematic review, therefore, aims to (1) define the prevalence of ED in people with anxiety disorders and (2) identify the severity of ED symptoms in this cohort.

## Methods

Preferred Reporting Items for Systematic Reviews and Meta-Analyses guidelines were followed when performing this review [11]. This review was also prospectively registered with PROSPERO (Registration Number: CRD42019161953).

### Study eligibility criteria

Inclusion criteria were studies investigating the erectile function of adults (age ≥ 18) male participants with a documented diagnosis of anxiety disorders made by at least one qualified psychiatrist. Anxiety disorders included were phobic disorders such as social anxiety disorder (SAD), panic disorder, obsessive–compulsive disorder (OCD), generalised anxiety disorder (GAD) and post-traumatic stress disorder (PTSD). Studies also required the use of a validated screening tool to measure erectile function or make a diagnosis of any type of ED, this includes organic and psychogenic. This included the International Index of Erectile Function-5 (IIEF-5) [12], Erection Hardness Score (EHS) [13] or Sexual Health Inventory for Men (SHIM) [14]. We also encompassed retrospective studies that utilised standardised diagnostic codes within databases such as ICD-10 [15] or DSM-IV [16].

We excluded studies with fewer than ten participants, case reports or expert opinions. We also excluded studies looking at rarer anxiety disorders, including adjustment disorders, dissociative disorders and somatoform disorders, as these were outside the scope of this review. Studies not published in the English language and conference abstracts were also excluded unless sufficient data or the full text was available.

### Information sources and search

We conducted a systematic search of the literature including all papers published before 28th November 2019 utilising the PubMed, Embase and PsycINFO electronic databases. The search terms devised were a mixture of key subject words and MeSH terms with the full strategy presented in Appendix 1. Grey literature was searched for unpublished studies using the International Standard Randomised Controlled Trials Number registry for any relevant ongoing studies, with authors of any applicable studies contacted for any preliminary data. If trials were identified which already had a publication, this was taken for inclusion to avoid duplication.

### Study selection

Titles, abstracts and subsequently full texts from the search results were screened by two reviewers independently (RV and MW). Discrepancies between reviewers after full-text review were resolved by a third reviewer (OB). A reference review of included studies was also conducted for any further pertinent articles. If full texts were not available the authors were contacted, and if still not available these studies were excluded.

### Data collection and data items

Data was extracted by the first author (RV) from each eligible study onto a pre-defined extraction sheet. Generic study data extracted included study characteristics such as first author, year of study, country of study, study design, total number of participants, the specific anxiety disorder investigated in the study and the criteria used to identify patients with anxiety disorder and ED. Additionally, we extracted specific data pertaining to the three outcome measures of this review: prevalence, severity and risk factors for ED. For the prevalence of ED in patients with an anxiety disorder, the number of participants with the anxiety disorder of interest and the number of those who had ED was extracted. In studies where the exact number of participants with ED and anxiety disorders was not reported, but the prevalence of ED and the number of participants with an anxiety disorder were, these values were used to calculate the rough number of participants with ED and anxiety disorders.

### Study quality assessment

Quality appraisal of individual studies included was done using the Risk of Bias in Non-randomised Studies of Exposures [17]. This tool evaluates confounders and assesses for risk of bias in seven domains in studies of different methodologies: confounding, participant selection, classification of exposure, departure from intended exposure, missing data, measurement of outcome and selection reported result. A score of low, moderate or serious was given for each domain, if a study received a serious score in at least one domain, it would automatically receive an overall score of serious. If it does not have any serious score and it received moderate scores in at least three different domains it will be given an overall score of moderate, otherwise, it will receive an overall score of low. A traffic light visualisation plot made using robvis shows the scores each study received on each domain [18].

## Results

### Study selection

**Fig. 1:**
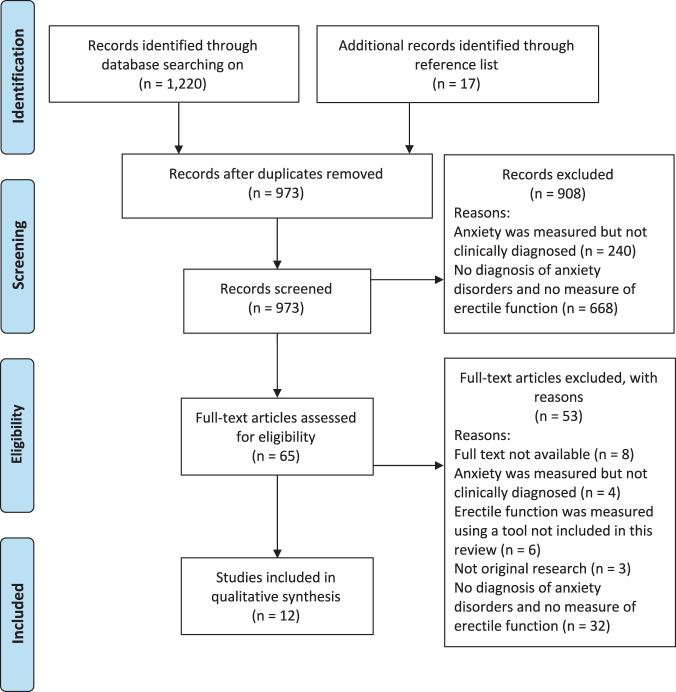
PRIMSA flowchart showing study selection. Flowchart showing the number of studies selected and excluded in each stage of the study selection process.

Our database search yielded 1220 articles for review. After removing duplicates, 956 were screened based on their titles and abstracts with a further 17 additional articles identified through reference review of the abstracts screened. Of the 65 articles that underwent full-text screening, 12 were included in this review (Fig. 1).

### Study characteristics

Of the 12 included studies, five were cross-sectional, three case–controls and four retrospective cohort studies. Six studies evaluated PTSD [19–24] and two OCD [25, 26], of which one also looked at SAD [25], and four looked at panic disorder [27–30]. Studies were published between 2001 and 2019 with a total of 713,746 participants. In all, 113,850 participants were diagnosed with an anxiety disorder investigated in this review by a clinician, with 111,091 diagnosed with PTSD, 33 with OCD, 2594 with panic disorder and for SAD it was 34. Sample sizes varied from 10 to 110,223. We also found that there were 406,616 veterans in the total study population across all studies.

Although we searched for studies which used IIEF-5, SHIM and EHS, all seven of the prospective studies that were selected used IIEF-5 to measure erectile function, whereas the remaining five retrospective studies use ICD-9 or DSM-IV codes to search the patient medical records to identify patients with ED. Detailed characteristics of all included studies are presented in Table 1.

**Table 1:** Characteristics of eligible studies including prevalence of ED in men with anxiety disorder.

Study	Anxiety disorder studied	Country	Study aims	Study design	Total male participants	Total male participates with anxiety disorders	No. of ED and AD participants	Prevalence	Anxiety disorder criteria (codes)	ED criteria	Limitation
Arbanas [19]	PTSD	Croatia	Comparing sexual functioning in patients with different intensity levels of PTSD and patients who receive treatment for PTSD	Cross-sectional	*n* = 164	*n* = 88			DSM-IV (309.81), CAPS	IIEF-5	All PTSD patients had combat PTSD; veteran population; PTSD sample may have secondary gain; exclusion of patients without partners.
Breyer et al. [20]	PTSD	USA	To find the prevalence and correlates of sexual dysfunction in a male veteran population	Retrospective cohort	*n* = 405,275	*n* = 110,223	*n* = 3356	3%	ICD-9 (F43.10), DSM-IV (309.81)	ICD-9	All retrospective data; veteran population; confounder of psychiatric medication use.
Breyer et al. [21]	PTSD	USA	Looking at the association between sexual functioning and PTSD in combat veterans	Retrospective cohort	*n* = 787	*n* = 512	*n* = 54	10.50%	ICD-9 (F43.10), DSM-IV (309.81)	ICD-9	All retrospective data; veteran population; all PTSD patients had combat PTSD.
Cosgrove et al. [22]	PTSD	USA	To evaluate the prevalence, correlates and severity of sexual dysfunction in a male veteran population	Case–control	*n* = 90	*n* = 44	*n* = 37.4^a^	85%	DSM-IV (309.81)	IIEF-5	Veteran population; confounder of psychiatric medication use.
Evren et al. [23]	PTSD	Turkey	To find the prevalence of PTSD in male alcohol-dependent patients and see if sociodemographic characteristics have an effect on the prevalence	Case–control	*n* = 82	*n* = 22	*n* = 17	77.30%	DSM-IV (309.81)	IIEF-5	All men had alcohol dependence; only had participants in relationships.
Letica-Crepulja et al. [24]	PTSD	Croatia	To assess the predictive models of sexual dysfunctions in male veterans with PTSD	Cross-sectional	*n* = 300	*n* = 300 (290^b^)	*n* = 134	46.20%	DSM-IV (309.81)	IIEF-5	Veteran population; missing data.
Fontenelle et al. [25]	OCD + SAD	Brazil	Compared sexual functioning in patients with OCD and SAD	Cross-sectional	*n* = 28	*n* = 13 (OCD) *n* = 15 (SAD)			DSM-IV (OCD = 300.3, SAD = 300.23)	IIEF-5	No controls; small sample size.
Ghassemzadeh et al. [26]	OCD	Iran	To evaluate sexual functioning in patients with OCD	Cross-sectional	*n* = 20	*n* = 20	*n* = 4	20%	MOCI, OCI-R, DSM-IV (300.3)	IIEF-5	Small sample size
Blumentals et al. [27]	PD	USA	To assess the relationship between panic disorder and ED	Retrospective case–control	*n* = 304,745	*n* = 1177	*n* = 427	36.20%	Found on IHCIS	ICD-9	All retrospective data; selection bias due to time restrictions on patient selection
Figueria et al. [28]	PD + SAD	Brazil	To evaluate sexual functioning in patients with social phobia and panic disorder	Retrospective cohort	*n* = 33	*n* = 14 (PD) *n* = 19 (SAD)	*n* = 1 *n* = 0	7.10% 0%	DSM-IV (PD = 300.01, SAD = 300.23)	DSM-IV	All retrospective data; small sample size
Okulate et al. [29]	PD	Nigeria	To find the prevalence of ED in men who were also screened for depression, alcohol abuse and panic disorder	Cross-sectional	*n* = 829	*n* = 10	*n* = 2	20%	PHQ, DSM-IV (300.01)	IIEF-5	All participants were military personnel; small sample size
Wang et al. [30]	PD	Taiwan	To explore the incidence rate of ED among panic disorder patients	Retrospective cohort	*n* = 1393	*n* = 1393	*n* = 28		ICD-9CM (300.01, 300.21)	ICD-9	All retrospective data

*CAPS* Clinician-Administered PTSD Scale, *DSM-IV* Diagnostic and Statistical Manual of Mental Disorders 4th Edition, *ICD-9* International Classification of Diseases 9th Revision, *IHCIS* Integrated Healthcare Information Services National Managed Care Benchmark Database, *IIEF-5* International Inventory of Erectile Function-5, *MOCI* Maudsley Obsessional-Compulsive Inventory, *OCD* obsessive–compulsive disorder, *OCI-R* Obsessive–Compulsive Inventory—Revised, *PD* panic disorder, *PHQ* Patient Health Questionnaire, *PTSD* post-traumatic stress disorder, *SAD* social anxiety disorder/social phobia.

^a^No. of ED with AD diagnosis was not reported, but we worked this out from prevalence and no. of AD.

^b^Prevalence calculation was not done using the whole AD male population (*n* = 300).

### Prevalence of erectile dysfunction in anxiety disorders

Ten studies provided data regarding the prevalence of ED in anxiety disorders [20–24, 26–30] (Table 1). The estimated prevalence of ED between the studies varied considerably, from 0.0 to 85.0% and the median [IQR] prevalence was 20.0 [5.1–41.2]%. Within the five studies that reported the prevalence of ED in PTSD participants, the range was 3.0–85.0% and the median [IQR] was 46.2 [10.5–77.3]% [20–24]. In four studies, the prevalence of ED in panic disorder was between 2.0 and 36.2% [27–30]. SAD and OCD both had one study each, which reported a prevalence of ED of 0.0% and 20.0%, respectively [26, 28].

### Severity of erectile dysfunction in anxiety disorders

All seven prospective studies used IIEF-5 to evaluate erectile function, the IIEF-5 is a self-report instrument to evaluate sexual functioning in males [12]. Depending on the score of the IIEF-5 the patient’s erectile impairment can be put into ED severity categories: no ED, mild, mild to moderate, moderate and severe [31]. Five of these studies reported the mean IIEF-5 scores for the erectile function domain (Table 2) [19, 23–26].

**Table 2:** Severity of ED in men with anxiety disorders.

Study	AD studied	Mean IIEF-5 scores (SD) of anxiety disorder groups	Diagnostic category of ED for anxiety disorder groups
Arbanas [19]	PTSD—treated	11.08 (9.19)	Moderate
	PTSD—untreated	11.76 (9.58)	Moderate
Evren et al. [23]	PTSD	18.30 (8.30)	Mild to moderate
Letica-Crepulja et al. [24]	PTSD	16.00 (9.71)	Moderate
Fontenelle et al. [25]	SAD	23.45 (8.26)	Mild
	OCD	17.62 (10.03)	Mild to moderate
Ghassemzadeh et al. [26]	OCD	25.80 (4.30)	Mild

The mean score from the five studies ranged from 11.08 to 25.80 with a median [IQR] score of 17.62 [13.88–20.88], which indicates a mild to moderate severity of ED. Most of the studies have a mean score indicating moderate or severe ED. One paper split the cohort of anxiety disorder participants into treated or untreated, reporting separate mean IIEF-5 [SD] of 11.08 [9.19] and 11.76 [9.58], respectively [19]. Another paper reported mean IIEF-5 scores for two different anxiety disorders, 17.62 [10.03] for OCD and 23.45 [8.26] for SAD [24].

Two of the PTSD studies looked at the correlation between PTSD symptom severity and ED severity [22, 24], both reported positive correlations between the two variables. In both studies, the PTSD symptom severity was measured using the DSM-VI symptom checklist for 309.81 [16].

### Risk of bias

**Fig. 2:**
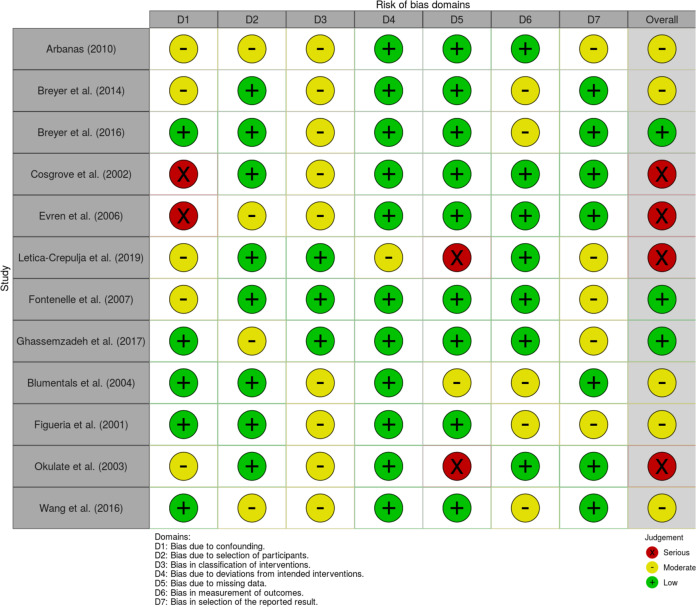
Risk of bias assessments summary for all studies. Traffic light plot showing risk of bias in each domain and the overall risk for all studies.

The summary of the risk of bias assessment for all the studies is presented in Fig. 2. No studies were found to have a critical risk of bias, however, 33.3% of studies had a serious risk and 41.7% had a moderate risk with the remaining 25% having a low risk. The main domains that were flagged to have a serious risk of bias, were biased due to confounding and missing data. The main confounders identified in the studies were alcohol abuse, marital status and use of psychotropic medications. In some studies, the whole anxiety disorder population was not used for the calculation for prevalence or mean IIEF-5 scores, these studies were flagged up for risk of bias due to missing data.

## Discussion

To our knowledge, this is the first systematic review to evaluate the prevalence of ED in anxiety disorders as a whole. Previous reviews have focused on a single anxiety disorder; however, we provide an overview of the spectrum of anxiety disorders [32, 33]. We identified a high prevalence of ED in patients with anxiety disorders, with a median value of 20%. There was, however, a wide range of estimates identified, likely secondary to the diverse methodology utilised in included studies. Additionally, this reflects the findings in the literature of the prevalence of ED as a whole, which varies widely, depending on age and how the diagnosis is made [33, 34].

Certain risk factors in the literature have been identified to make this population of patients more likely to suffer from ED. This includes the use of psychotropic drugs such as selective serotonin reuptake inhibitors. It has been shown that antidepressants like these may cause 30 to 40% of patients to develop sexual dysfunction [35]. Another risk factor is the co-existence of other psychiatric conditions with anxiety disorder, this was especially a concern in PTSD, as studies have shown that patients with PTSD usually have an additional mental health diagnosis [36]. Depression is the most common co-morbid psychiatric condition and depressive symptoms have been associated with ED, a meta-analysis found that the pooled odds ratio for risk of ED in depression exposure was 1.39 (95% CI: 1.35–1.42) [8]. The overlie between anxiety and depressive symptoms could not be fully investigated in this review due to the inability to identify where this overlap was. An additional risk factor is increased alcohol consumption or alcohol abuse; it has been shown that alcohol’s depressive effect also gives short-term alleviation of anxiety disorder symptoms, so patients not receiving adequate treatment may resort to self-medication through alcohol which may lead to abusive tendencies [37]. The same depressive effect of alcohol is thought to contribute to the development of ED [38].

Moreover, the prevalence of ED in the different anxiety disorder subgroups varied immensely, we found the prevalence in the PTSD population to range between 3 and 85%, this could be because the studies looking at PTSD had a sample size varying from 82 to 405,275. Bentsen et al. [32] conducted a systematic review on sexual dysfunction in veterans with PTSD and found the prevalence of ED was between 63 and 85%.

A meta-analysis, with a total sample of 48,254, reported that the severity-specific prevalence of ED in mainland China was 32.54%, 9.86% and 13.97% for mild, moderate and severe ED, respectively, suggesting that more people in the general population of China with ED had a mild severity [39]. Other individual studies with large sample sizes from other parts of the world also report that mild ED is the most common severity type [40, 41]. The median severity of ED reported by the studies in the review was 17.62, indicating a mild to moderate severity. This could suggest anxiety disorder patients may be at risk of developing ED at a higher severity than the general population, however, due to the sizable heterogeneity between the studies in this review this association cannot be implied.

The findings of this review suggest that the anxiety disorder populations are at a higher risk of developing ED. The role of anxiety in sexual functioning in this population has not been clearly established but it is thought that an abnormal anxiety response causes an increase in sympathetic tone, resulting in a distraction from erotic stimuli leading to impaired arousal and erection [4]. Therefore, in psychiatric practice and in primary care clinicians must routinely screen for sexual dysfunction in patients with anxiety disorders and refer then to urology to attain the right support they need, especially if they exhibit some of the common risk factors of both conditions such as using psychotropics. In this case, clinicians should evaluate baseline erectile function before commencing anxiety disorder patients on any psychotropic medications and adjust the dosages appropriately, this could increase adherence to the medication, while not compromising their quality of life [42]. Overall, the significance of the findings in this review is that caring for patients with ED and anxiety requires a multidisciplinary approach requiring psychiatric clinicians to work together with the urology team to attain the best outcomes for the patient.

### Limitations

The findings of this review cannot be generalised to the universal population of men with anxiety disorders, as 57% of our sample size were veterans, who are a population that exhibit many predictors for ED. Therefore, this does bias our assumptions and our findings may be an overestimate of the true association between ED and anxiety disorders, if we excluded studies involving veterans, we may have had more reliable findings. Furthermore, the most common anxiety disorders in the general population are phobic disorders, SAD and GAD [43], which were tremendously underrepresented in our review. This could be because there are not many studies that look at ED in this population or it could be due to our search strategies, as we only included studies published in the English language consequently missing out a large proportion of the literature. While going through the study screening process, we found many studies that measure anxiety levels but do not actually diagnose an anxiety disorder. Many of these studies used the Generalised Anxiety Disorder scale (GAD-7) [44] to quantify GAD symptoms but do not clinically diagnose GAD. In addition, in many countries clinical psychologist commonly diagnose mental health conditions besides psychiatrists, if we included these studies and the studies that used the GAD-7, we may have had a study population with better represents the general population.

From the literature, we know that the prevalence of ED increases with age [38]. Therefore, many studies report the age-specific prevalence. However, we were not able to find the mean ages of most of our studies, as they were calculated for the whole study population which included female participants or participants without anxiety disorders. In addition, a third of the studies in this review were flagged as high risk of bias. The presence of these biases makes it seem like there is an association between having anxiety disorders and ED or even mask a true association. Also, we were not able to distinguish the different types of ED in the study population to see if anxiety has a different role to play in organic and psychogenic ED.

Further research must be undertaken to find the true association between ED and anxiety disorders, this should focus on each subgroup of anxiety disorders rather than anxiety disorders as a whole. This is because the prevalence of ED in each subgroup varies drastically, this could be due to different risk factors in each subgroup, for example, military deployment and experiencing combat trauma were recognised as potential risk factors identified in the PTSD studies.

## Conclusion

Our review identified a high prevalence of ED in the anxiety disorder population and evidence that suggest ED may be more severe in this population, therefore this advocates that this is an important clinical topic. However, the evidence is limited because of the high heterogeneity between the study populations of the papers. More research is required to help improve patient care in this population. Literature suggests that men with anxiety disorders may exhibit risk factors for ED, but it is hard to say whether anxiety disorder alone is a risk factor for ED. For future research, it would be good to eliminate potential confounders and risk factors of ED, therefore, using treatment-naive populations with a singular psychiatric diagnosis of an anxiety disorder in a cross-sectional study measuring ED using a validated tool like the IIEF-5 on a large sample of anxiety disorder participants encompassing these factors mentioned earlier will give a good idea about the true prevalence and severity of ED in this population.

## Supplementary information

Search strategy
