# Supplementary material for: Erectile dysfunction in patients with anxiety disorders: a systematic review
Source: Int J Impot Res. 2021 Feb 18;34(2):177–86. doi: 10.1038/s41443-020-00405-4 (PMC8964411; doi:10.1038/s41443-020-00405-4)
Supplement: Supplementary file 1 — Search strategy [file 41443_2020_405_MOESM1_ESM.pdf]

Appendix 1 - Full search strategy utilised

<u>Search strategy</u>

- PudMed Search
    - (Anxiety disorders OR Neurotic Disorders OR Social Phobia OR Anxiety OR Generalised anxiety disorder OR GAD OR Obsessive-compulsive disorder OR OCD OR neurosis OR Panic disorder OR post-traumatic stress disorder OR PTSD) AND (erectile dysfunction OR Impotence OR erectile function OR ED OR Sexual dysfunction OR psychosexual dysfunctions) AND (International Index of Erectile Function OR IIEF* OR Erection Hardness Score OR EHS OR Sexual Health Inventory for Men OR SHIM)

- Embase Search (via Ovid)
    - ((Anxiety disorders OR Neurotic Disorders OR Social Phobia OR Anxiety OR Generalised anxiety disorder OR GAD OR Obsessive-compulsive disorder OR OCD OR neurosis OR Panic disorder OR post-traumatic stress disorder OR PTSD) AND (erectile dysfunction OR Impotence OR erectile function OR ED OR Sexual dysfunction OR psychosexual dysfunctions) AND (International Index of Erectile Function OR IIEF* OR Erection Hardness Score OR EHS OR Sexual Health Inventory for Men OR SHIM)).af.

- PsycINFO (via Ovid)
    - ((Anxiety disorders OR Neurotic Disorders OR Social Phobia OR Anxiety OR Generalised anxiety disorder OR GAD OR Obsessive-compulsive disorder OR OCD OR neurosis OR Panic disorder OR post-traumatic stress disorder OR PTSD) AND (erectile dysfunction OR Impotence OR erectile function OR ED OR Sexual dysfunction OR psychosexual dysfunctions) AND (International Index of Erectile Function OR IIEF* OR Erection Hardness Score OR EHS OR Sexual Health Inventory for Men OR SHIM)).af.
